# Overview of methods for characterization and visualization of a protein–protein interaction network in a multi-omics integration context

**DOI:** 10.3389/fmolb.2022.962799

**Published:** 2022-09-08

**Authors:** Vivian Robin, Antoine Bodein, Marie-Pier Scott-Boyer, Mickaël Leclercq, Olivier Périn, Arnaud Droit

**Affiliations:** ^1^ Molecular Medicine Department, CHU de Québec Research Center, Université Laval, Québec, QC, Canada; ^2^ Digital Sciences Department, L'Oréal Advanced Research, Aulnay-sous-bois, France

**Keywords:** interactome, biological network, computational prediction, integrated strategies, graphic view, protein-protein interaction

## Abstract

At the heart of the cellular machinery through the regulation of cellular functions, protein–protein interactions (PPIs) have a significant role. PPIs can be analyzed with network approaches. Construction of a PPI network requires prediction of the interactions. All PPIs form a network. Different biases such as lack of data, recurrence of information, and false interactions make the network unstable. Integrated strategies allow solving these different challenges. These approaches have shown encouraging results for the understanding of molecular mechanisms, drug action mechanisms, and identification of target genes. In order to give more importance to an interaction, it is evaluated by different confidence scores. These scores allow the filtration of the network and thus facilitate the representation of the network, essential steps to the identification and understanding of molecular mechanisms. In this review, we will discuss the main computational methods for predicting PPI, including ones confirming an interaction as well as the integration of PPIs into a network, and we will discuss visualization of these complex data.

## Introduction

Proteins are essential to life, controlling molecular and cellular mechanisms. Their main role is to carry out cellular biological functions through interactions with molecules or macromolecules (Pellegrini et al., 1999; Vinayagam et al., 2014; Fionda, 2019). These interactions are organized in networks (Bersanelli et al., 2016) of various molecular elements (e.g., protein–DNA and protein–drug) involved in physical and biochemical processes in structured environments. Biological networks have been highlighted by the work of Barabási and Oltvai (2004), who showed that cellular networks are governed by universal laws. This new concept revolutionized the vision of system biology, initiating creation and analysis of the first protein–protein interaction (PPI) network of yeast *Saccharomyces cerevisiae* (Dezso, Oltvai and Barabási, 2003).

In the PPI network, proteins are represented by nodes, and interactions between proteins by edges (Gursoy, Keskin and Nussinov, 2008; Zou et al., 2018). The size of the network and the amount of information (e.g., discovered node) varies between species (Kotlyar, Rossos and Jurisica, 2017; Wang and Jin, 2017). The number of PPIs is constantly changing due to complexity of the genome and many interactions remain undiscovered (Safari-Alighiarloo et al., 2014; Thanasomboon et al., 2020). PPIs can be determined by high-throughput experiments such as co-immunoprecipitation, two-hybrid screening, pull-down assays (MacDonald, 1998; Lin and Lai, 2017; Louche, Salcedo and Bigot, 2017), or by computational methods. Experimental methods are time-consuming, relatively expensive, and difficult to reproduce (von Mering et al., 2002; Piehler, 2005; Browne et al., 2010; Ngounou Wetie et al., 2013). In response to these challenges, computational methods have emerged, showing promising results in terms of performance to integrate functional (i.e., same biochemical reaction) and physical interactions. A physical interaction describes a physical contact between proteins, as a result of biochemical events steered by interactions including electrostatic forces, hydrogen bonding, and the hydrophobic effect (Berne, Weeks and Zhou, 2009; Nitzan, Casadiego and Timme, 2017). These computational methods allow a more specific identification of interactions than experimental prediction methods (Droit, Poirier and Hunter, 2005; Shoemaker and Panchenko, 2007; Zhou, Li and Wang, 2016).

Although PPIs from computational methods provide a better prediction of physical interactions, PPI databases contain a few false positive interactions (Peng et al., 2017; Luck et al., 2020). One way to remove these false interactions is through integration methods (as can be seen in session integration of a PPI network). Following the integration of the data, it becomes possible to filter PPI. To observe the resulting network and the proteins having a role in mechanisms, visualization is a key step.

Visual representation allows to understanding PPIs and to analyze networks (Iranzo, Krupovic and Koonin, 2016; Armanious et al., 2020; Schneider et al., 2021; Sejdiu and Tieleman, 2021). However, due to complexity of proteomes of different organisms, visualization is a challenge (Crowther, Wipat and Goñi-Moreno, 2021). Moreover, the density of the graph representing the proportion of interactions in the network compared to the total number of possible interactions makes representation more difficult (Ren et al., 2013; Franzese et al., 2019; Wu et al., 2019). To facilitate representation, the network is divided into sub-networks (He and Chan, 2018; Farahani, Karwowski and Lighthall, 2019). These sub-networks are obtained by filtration or by decomposing the network according to proteins of interest, with the concept of ego network (Liu et al., 2019; Tian, Ju and Yang, 2019). Ego networks are subgraphs centered on a seed node and comprise all nodes connected at a defined distance from the ego (seed node) (Zhou, Miao and Yuan, 2018; Malek, Zorzan and Ghoniem, 2020). Sub-networks facilitate representation and allow identification and understanding of cellular mechanisms, core proteins, or biomarkers (Gehlenborg et al., 2010; Laniau, 2017; Hao et al., 2019).

In this review, we will discuss computational methodologies for construction of PPI networks as well as integration and validation of these networks. Next, we will discuss the visualization aspect of a network by discussing its roles and advantages and disadvantages of different visualization tools.

### Computational methods for PPI construction

Computational methods for predicting PPIs can be classified into three prediction methods: based on the genomic context, machine learning algorithm, and text mining (Table 1).

**TABLE 1:** Summary table of computational methods for the prediction of a protein–protein interaction. Computational methods for predicting PPIs are grouped into three distinct categories: genomic context–based methods, machine learning, and text mining. Within each of these approaches, several sub-methods exist. A database can be composed of interactions obtained by several prediction methods.

	Main method	Main advantage	Main disadvantage	Database
Genomic context	Domain fusion, conserved gene neighborhood, phylogenetic profiles, and co-evolution (De Las Rivas and Fontanillo, 2010; Raman, 2010; Rao et al., 2014)	Interspecies comparison requires few IT resources, fast calculation	Low coverage rate, prediction, using only genomic features	String (Szklarczyk et al., 2019), BioGRID (Oughtred et al., 2021), Hippie (Alanis-Lobato, Andrade-Navarro and Schaefer, 2017), IntAct (Hermjakob et al., 2004a), HPRD (Keshava Keshava Prasad et al., 2009)
Machine learning algorithm	Supervised learning: support vector machine, artificial neural networks, naïve Bayes learning, decision trees (Sarkar and Saha, 2019; Chakraborty et al., 2021)	Handling multi-dimensional and multi-variety data, high efficiency	Data acquisition (massive datasets), High error susceptibility, requires significant IT resources	String, BioGRID, IID (Kotlyar et al., 2019), Hitpredict (Patil, Nakai and Nakamura, 2011)
	Unsupervised learning: K-means, hierarchical clustering (Bello-Orgaz, Menéndez and Camacho, 2012; Lu et al., 2021)			
Text mining	Extracting information from scientific studies and references databases as PubMed	Many publications are available, rapidity of execution, inexpensive, easily accessible data	Requests that the interactions be cited in the articles	String, BioGRID, MINT (Chatr-aryamontri et al., 2007), IntAct, HPRD (Keshava Prasad et al., 2009)
	Using natural language processing (NLP) technology			
	(Raja, Subramani and Natarajan, 2013; Vyas et al., 2016; Badal, Kundrotas and Vakser, 2018)			

The methods can be combined to refine the prediction of PPIs. Alachram et al. (2021) exploited text mining algorithms mixed with machine learning algorithms to capture biologically significant relationships between entities, including PPIs.

## Methods based on genomic context

The genomic context refers to the structure of genomic data (e.g., genes), as well as the statistical or mathematical methods to test for gene, protein set association (Dimitrieva and Bucher, 2012; Mooney et al., 2014). Genomic context methods are usually based on gene sequences, structure, and organization of genes on the chromosome (Skrabanek et al., 2008; De Las Rivas and Fontanillo, 2010; Reimand et al., 2012; Rao et al., 2014).

### Domain fusion interaction prediction method

Gene fusion leads to fusion proteins, which are an assembly of several proteins encoded by different genes created by joining (fusion) of one or more genes (Morilla et al., 2010; Latysheva et al., 2016). This fusion results in a single or multiple polypeptides that takes on the functional properties of each in original proteins. The existence of a functional interaction between protein A and protein B is based on the hypothesis that if protein domains A and B of one species have fused homologs in a single AB polypeptide in another species, then domains A and B are functionally linked (Truong and Ikura, 2003; Chia and Kolatkar, 2004). The gene fusion method marked a major turning point in methods for predicting PPIs. This computational method, developed by Eisenberg et al. (2000), was the first computational method to find PPIs from the genome of distinct species based on polypeptides (Marcotte et al., 1999).

The comparison of inter-species sequences can show AB sequences, which are also called Rosetta stones because they allow the interaction between A and B to be deciphered (Date, 2007). This method assumes that if the affinity of A and B increases as B increases when A is fused to B, then pairs of proteins may have evolved from proteins with A and B interaction domains on the same polypeptide (Chia and Kolatkar, 2004; Kamisetty et al., 2011). To improve this method, Veitia, (2002) integrated eukaryotic gene sequences. This incorporation increases robustness of AB polypeptide prediction due to the larger volume of sequences in eukaryotes. A question of equilibrium explains this increase in robustness: the required concentrations of proteins A and B cannot be higher than the equilibrium concentration of AB polypeptides, proteins A and B cannot be separated. Despite the addition of these sequences, few PPIs are found explaining a limited interactome or many PPIs are missing (Latysheva et al., 2016). This method is usually combined with other methods such as machine learning methods (De Braekeleer, Douet-Guilbert and De Braekeleer, 2014; Birtles and Lee, 2021). The accuracy values, therefore, take several methods and are not specific to the domain fusion method. Tagore et al. (2019) have developed the ProtFus tool which combines machine learning, protein fusion, and text mining methods to obtain accuracy values between 75% and 83% to predict PPIs.

### Conserved gene neighborhood

This method relies on neighbor gene conservation at the genomic scale. This method compares the position of genes from different genomes to predict potential interactions (Dandekar et al., 1998). For example, a gene is always next to the B gene. Two direct neighboring genes in different genomes suggest interactions. This method is widely used in the prediction of PPIs in eukaryotes (Rogozin et al., 2002). Nomenclature discrepancies in ortholog genes, as well as the search of orthologs that are adjacent on chromosome, explain the low predictive coverage of PPIs (Raman, 2010; Lv et al., 2021). Recently, this method in multi-omics integration has confirmed that bacterial genomes are not randomly organized and can form clusters depending on the local genomic context (Esch and Merkl, 2020). They obtained an accuracy value of 55%. As they mention, this type of method is not intended for the discovery of direct interactions. Recently, a new tool: GENPPI (Anjos et al., 2021), allowing the generation of PPI networks by taking into account evolutionary relationships that can only be annotated from genomes, namely, conserved gene neighborhoods (CN), phylogenetic profiles (PPs), and gene fusions, has been introduced, showing that these three methods mainly allow the annotation of missing data and thus the understanding of a limited number of interactions. At present, the tool is being tested in their laboratory.

### Phylogenetic profiles

This method is based on the comparison of phylogenetic data between gene families of different organisms (Pellegrini et al., 1999; Škunca and Dessimoz, 2015). The phenotypic profile is represented by a binary vector composed of values 0 and 1, corresponding to the absence and presence of proteins in an organism, respectively. Proteins with close or similar phylogenetic profiles tend to be strongly functionally related (Pellegrini, 2019). Ding and Kihara (2018) recently implemented this approach to predict new interactions from known *Arabidopsis thaliana* interactions. The phylogenetic profile approach is combined with machine learning approaches. This method allowed the detection of PPIs with high precision and accuracy. In their work, the performance values range from 75% to 93.2% accuracy.

### Coevolution

Coevolution is a fundamental principle of evolutionary theory. Coevolution is defined as the chain of transformation events during the evolution of two species in a mutually dependent manner (de Juan, Pazos and Valencia, 2013). Coevolution results from selective pressure between two or more species (Anderson and de Jager, 2020; Takagi et al., 2020). The interactions of coevolved proteins can be kept either by direct binding or by functional associations (Tillier and Charlebois, 2009). If there is an interaction between two proteins, when one protein mutates, the other protein might have a compensatory mutation, otherwise; two proteins cannot support stability or functions of the interaction during evolution. The evolutionary pressure resulted in the elaboration of co-evolutionary protein pairs in cells that keep the interaction and therefore the function of the protein (Pazos et al., 1997; Goh and Cohen, 2002; Xia et al., 2008).

The global advantage of methods based on the genomic context is the interspecies comparison that requires high computing resources (Sun et al., 2008; Pattin and Moore, 2009). The limitations of these methods are a limited number of predicted PPIs, using only genomic features (Chiang et al., 2007; Raman, 2010; Rao et al., 2014). Recent work by Green et al. (2021) using coevolution had accuracy values of the order of 80% showing promising results for the prediction of protein interaction structures and interfaces. The work of Croce et al. (2019) offered similar results in terms of accuracy for the prediction of protein domain interactions.

The methods based on the genomic context are relevant for evolutionary history analysis, small proteome size, or for experimental verification, agronomic analysis on mutations, or other variants (Koh et al., 2012; Zahiri, Bozorgmehr and Masoudi-Nejad, 2013; Malik, Sharma and Khatri, 2017). On the other hand, these prediction methods are less appropriate for medical data analysis, especially for the search of driving proteins in mechanisms due to the high complexity of the human proteome (Kuzmanov and Emili, 2013; Zhong et al., 2019; Swamy, Schuyler and Leu, 2021).

## Methods based on the machine learning algorithm

Machine learning (ML) belongs to the field of artificial intelligence (AI) and computer science. ML algorithms learn from already obtained data to predict outcomes in a specific context (El Naqa and Murphy, 2015; Murdoch et al., 2019). This field has undergone a considerable revolution in the last 10 years with the emergence of promising new methods for PPI prediction (Ding and Kihara, 2018; Kotlyar et al., 2019; Das et al., 2020). ML can be classified into two subclasses: supervised and unsupervised learning. Supervised learning can be defined as a machine learning task that learns to predict from labeled data, conversely; unsupervised learning will learn to predict an outcome on unlabeled data (Zhao, Wang and Wu, 2017; Sarkar and Saha, 2019; Razaghi-Moghadam and Nikoloski, 2020).

### Supervised learning method for PPI prediction

#### Support vector machines

Support vector machines, developed by Vapnik, (1963); (Cortes and Vapnik, 1995), build the best hyperplane to separate training sample classes by a maximal margin, with all positive samples lying on one side and all negative samples lying on the other side. Hyperplane, in the framework of a PPI network, will classify the protein pairs as a binary problem. Protein pairs serve as input, and it classifies if an interaction is possible or not. Protein pairs that are close to the hyperplane are called support vectors and predict an interaction between that pair of proteins (Sarkar and Saha, 2019; Chakraborty et al., 2021).

Ma et al. (2020) developed a method called ACT-SVM for predicting PPIs. This model maps protein sequences to numerical features. Extraction of numerical features is performed twice on the protein sequence to obtain two vectors: a vector and descriptor CT (composition and transformation) are combined to form a single vector. Feature vectors of a protein pair will be the input of the SVM. The closer these feature vectors of a pair of proteins are to to hyperplane, the higher the probability of an interaction between these proteins.

Dunham and Ganapathiraju, (2021) benchmarked different PPI prediction algorithms, and show how well they perform on realistically proportioned datasets. Based on verified interactions and a known false interaction rate, 16 datasets using the SVM method are generated. Accuracy values ranged from 51 to 96%, which highlights false interactions predicted or not predicted by the SVM methods.

#### Artificial neural networks

Artificial neural networks (ANNs) are inspired by neural networks in the brain (Wang, 2003; Zhang, 2018). An artificial neural network is composed of different layers with a variable number of neurons, and each layer is connected between them (Yann Lecun, 1986). To simplify, an ANN network works like an artificial neuron that can receive and send information as a signal to the neurons connected to it. This signal is represented by a real number calculated by a non-linear function of the sum of the inputs to a neuron. Neurons and edges can be weighted, and the weighting is adjusted during the learning process. Weight varies according to the intensity of the signal. Signals travel from the first to the last layer, and this results in the output of active neurons (those with a high intensity) (Baxt, 1995; Krogh, 2008; Dongare, Kharde, and Kachare, 2012).

In the context of PPI prediction, artificial neurons represent pairs of proteins. The signal propagates between different artificial neurons. Neurons and edges with high intensity suggest a connection between proteins. A suggested input for these algorithms is the protein sequences of two proteins, other inputs can be put such as 3D structures of proteins (Xie, Deng and Shu, 2020; Pan et al., 2021). The prediction of PPIs based on their amino acid sequences as well as their physiochemical properties is of great interest to understand the probabilistic constraints of the prediction (Ahmed, Witbooi and Christoffels, 2018; Tang et al., 2021). Sharma and Shrivastava (2015) applied an ANN approach that takes the animated acidic sequences of protein pairs as inputs and returns as output whether the pair interacts or not.

The ANN method had quite similar results to the SVM methods. The accuracy values are variable, Hu et al. (2021) showed an accuracy of 71.5% for the prediction of hot spots in a PPI while Pan et al. (2022) observed an accuracy of about 90% in predicting protein interactions in *Arabidopsis thaliana* as a result of this work.

ANNs are exploited as a reference method in several classification tasks (Rohani and Eslahchi, 2019; Baek et al., 2021), but they suffer from some limitations. Artificial neurons that are interaction pairs are checked to limit the introduction of bias during the prediction step (H. Li et al., 2018a; Wu et al., 2021).

#### Naïve Bayes classifier

A naïve Bayes classifier (NBC) relies on the simple probability of the Bayes’ theorem (Bayes et al., 1763). NBC classifies an item by taking each feature of the item independently (e.g., color and shape). To predict a PPI interaction, protein sequences are split into several sub-sequences of n residues. Bayes classifier establishes a probability matrix allowing to classify the different residues; residues that will interact with each other and the non-interface residues. This method is based on conditional probabilities, the probability that is an interaction knowing that an interaction has already occurred. This method will predict interaction sites from protein sequence information alone (Murakami and Mizuguchi, 2010; Geng, Chen and Wang, 2021). Accuracy values are generally lower than those of the SVM and ANN methods, due to the difference in the amount of information available on the proteins.

In PPI prediction, each observation is represented by a vector Z (X_1_; X_2_;X_3_;….; X_m_,Y), where X{X1,X_2,_X_3,_….,X_m_} is the m-dimensional input variable and Y is the output variable taking {0,1}. As input, this method can take either protein interaction datasets or genomic interaction datasets (Jansen et al., 2003; Alashwal, Deris and Othman, 2009; Lin et al., 2021). In the end, the classifier gives a binary response, a zero indicating the interaction is not verified, and a one when there is a potential interaction. Geng et al. (2015) adopted naive Bayes classification to predict site interactions between two proteins. Each pair of proteins is split into several residues, with two residues of two proteins in the same cluster interacting. In terms of performance, they achieved an accuracy value of 60%, which is generally lower than those of the SVM and ANN methods, due to the difference in the amount of information available on the proteins (Ahmed, 2020; Jonathan et al., 2021; Lin et al., 2021).

Identification of interface residues by this method is less expensive and gives results comparable to experimental methods for the prediction of interactions (Murakami and Mizuguchi, 2010; Amirkhah et al., 2015).

### Decision trees

A decision tree is a statistical tool that will represent a set of choices as a hierarchical tree. According to different choices made, the algorithm ranks the input elements according to distinctive features: domain presence, spatial folding, site fixation, etc. The decision tree will classify the pair of proteins either as interacting (the proteins in the pair interact with each other) or as non-interacting. Each pair of proteins is characterized by several information and subdomains forming a vector. An interaction is predicted as true if the probability of interactions between two different protein domains is high (Chen and Liu, 2005).

Lee and Oh, (2014) exploited the decision tree method to find discriminating biological features that allow the identification and identify true positive interaction. They have acquired accuracy averages of 97%. This classification helps to understand the biological context of an interaction. The performance of these methods is dependent on the amount of information available for a biological entity and the projection of low-dimensional features (Xuan et al., 2019; Blassel et al., 2021; Zhou et al., 2021). Li et al. (2021) presented challenges of these methods in terms of performance.

Within supervised methods, a sub-class of methods has emerged in recent years: self-supervised learning methods (Chen et al., 2022; Murphy, Jegelka and Fraenkel, 2022), able to train themselves to learn and predict the output of one part of the input data from another part of the data (Wang et al., 2021; Guo et al., 2022). A graph neural network is a self-supervised method for predicting interactions and in particular PPIs (Mahdipour and Ghasemzadeh, 2021; Jha, Saha and Singh, 2022; Y. Wu et al., 2022b). They are based on machine learning algorithms that extract important information from graphs and use this information to make predictions (Li et al., 2020b; Shen et al., 2021). Jha, Saha, and Singh (2022) developed a method for predicting PPI interactions based on structural information contained in the PDB (Burley et al., 2021) and the sequence characteristics of proteins. The molecular graph of a protein has nodes representing the amino acids (also called residues) of which proteins are made up of. A PPI is formed when pairs of atoms contained in two different residues, have a Euclidean distance less than the threshold distance set, here 6 angstroms. They obtained accuracy values after training of 99.5%. The results of this work show better prediction effectiveness than traditional machine learning methods such as SVM and ANN. Although this method is recent, the resulting accuracy values for interaction prediction are promising such as the prediction of drug–target interactions with an average accuracy value of 89.76% (Zhao et al., 2021), and the prediction of ncRNA–protein interactions with an accuracy value of 93.3% (Shen et al., 2021).

### Unsupervised learning method for PPI prediction

The unsupervised analysis includes several methods. The most widely used method is clustering, which aimed to group data into clusters. We will focus on two main clustering methods in the context of creating PPI networks (Malouche, 2013; Creusier and Biétry, 2014).

### Clustering methods

K-means clustering and hierarchical clustering methods are unsupervised learning techniques, the most used in the prediction of PPIs (Johansson-Åkhe, Mirabello and Wallner, 2019; Nath and Leier, 2020; Wang et al., 2020; Shirmohammady, Izadkhah and Isazadeh, 2021). Proteins will be clustered according to common characteristics (Ou-Yang, Yan and Zhang, 2017). Clustering steps are repeated to refine the clusters and improve prediction of PPIs (Bello-Orgaz, Menéndez and Camacho, 2012; Lu et al., 2021). Proteins in the same cluster have a high probability of interaction (Geng, Chen and Wang, 2021).

The input data can be of various nature for the prediction of PPIs (Krause, Stoye and Vingron, 2005; Zhao, Wang and Wu, 2017; Wang et al., 2020). Sun et al. (2008) relied on the phylogenetic profile of a protein as input. The phylogenetic profile is a comparative genomic method that predicts the large-scale biological molecule function through evolution information (Mikkelsen, Galagan and Mesirov, 2005). Liu et al. (2018) resorted to hot spot residues databases and in particular the Alanine Thermodynamic Scanning Database. Hot spot residues are functional sites in protein interaction interfaces, and these sites allow the understanding of the type of interactions and are highly conserved in proteins to ensure the functions. Itraq (K. Wang et al., 2018a) used protein sequences as input and hierarchical clustering to identify age-related biomarkers of dental caries. Protein interactions were then successfully validated by multiple reaction control mass spectrometry.

Each of these two clustering methods has sub-methods. For example, hierarchical clustering methods can be divided into two sub-families: “bottom-up” and “top-down” methods (Maimon and Rokach, 2006; Wang et al., 2010; S Bhowmick and Seah, 2015).

Clustering methods are known to be sensitive to noisy data due to experimental bias during acquisition of protein sequences (Arnau, Mars and Marín, 2005; Brohée and van Helden, 2006; Wang et al., 2008). As a result, false-positive interactions appear in the clusters (Sloutsky et al., 2013; Pizzuti and Rombo, 2014; Aghakhani, Qabaja and Alhajj, 2018; Stacey, Skinnider and Foster, 2021).

The global advantage of methods based on machine learning is the processing of multidimensional and multivariate data from several omics or horizontal omics (Das et al., 2020; Jamasb et al., 2021). Prediction of interactions is highly efficient (Terayama et al., 2019; Balogh et al., 2022), but machine learning requires large computational resources and large datasets of good quality (Hashemifar et al., 2018; Y. Wang et al., 2018b).

Machine learning–based approaches are approaches that will be scalable in different domains, these approaches offer very promising results (Casadio, Martelli and Savojardo, 2022; Huang et al., 2022; Pan et al., 2022). However, as we have seen in the articles, many sequences or interactions are necessary to train the model (Li M. et al, 2022; Hu et al., 2022; Jha, Saha and Singh, 2022). So, these approaches will be preferred for large-scale omics approaches, prediction of new interactions, or identification of clusters or hubs (protein with many interactions) (Pei et al., 2021; Song et al., 2022; Stringer et al., 2022). Different studies on PPI by You et al. (2013), Shirmohammady, Izadkhah, and Isazadeh (2021), and Kusuma et al. (2019), respectively, showed an accuracy of 88%, 63.8%, and 84.6% for clustering methods. This difference in accuracy is explained by the fact that clustering methods depend on the annotations and missing data contained in them (Wang et al., 2010; Zhou et al., 2022).

## Methods based on text mining

Text mining is a technique for exploring and transforming unstructured text into structured data (e.g., tables). In PPI prediction, text mining allowed to extracting information about proteins and their interactions from scientific studies and reference databases. Text mining techniques try to automate the extraction of sentence-related proteins from abstracts or paragraphs of text corpora (Papanikolaou et al., 2015). Several text mining methods exist, some are based on statistical matches between gene names, protein names in public repositories, and online resources. Links and types of interactions between proteins are defined by action verbs, for example, interact, interfering, and reacting. He, Wang and Li (2009) benefited from this technique through the PPI finder tool that was developed to extract human PPIs from PubMed abstracts based on their co-occurrences and interaction words, the retrieved interactions are then validated by the occurrence of Gene Ontology (GO) terms. More complex text mining methodologies use advanced dictionaries and generate natural language processes (NLPs) to build networks. The networks generated by these methods have as nodes the names of the genes or proteins, and as edges the verbs found. By these methods, a semantic notion is added (Raja, Subramani and Natarajan, 2013; Badal, Kundrotas and Vakser, 2018; Roth, Subramanian and Ganapathiraju, 2018). Newer methods utilized kernel methods, a class of algorithms for pattern analysis, to predict PPIs from the text. Vyas et al. (2016) applied this method and data mining for disease-related protein identification, functional annotation, and other proteomic studies. The overall advantage of text mining–based methods is the amount of information available and the extremely low cost to acquire PPIs (Alanis-Lobato, 2015; Zhu and Schmotzer, 2017). The main limitation is that the interactors must be close together or in the same sentence (Badal, Kundrotas and Vakser, 2015; Bajpai et al., 2020). Text mining methods have generally high accuracies because PPIs come from the text published as a result of experiments, thus reducing false interactions. For example, the InfersentPPI (Li X. et al, 2022) tool gave an accuracy value of 0.89 for humans, and the ModEx (Farahmand, Riley and Zarringhalam, 2020) tool gave an assurance value of 0.88.

Interaction prediction methods based on text mining are highlighted in the literature because of the large amount of data available in all domains (Jia et al., 2018; Khashan, Tropsha and Zheng, 2022). These methods are recommended for the study of molecular mechanisms and for a large and fast statistical analysis. But in the context of new experiments where little information is available, these methods do not seem to be very suitable (Elangovan, Davis and Verspoor, 2020; Piereck et al., 2020; Shi et al., 2021).

### Integration of a PPI network

**FIGURE 1:**
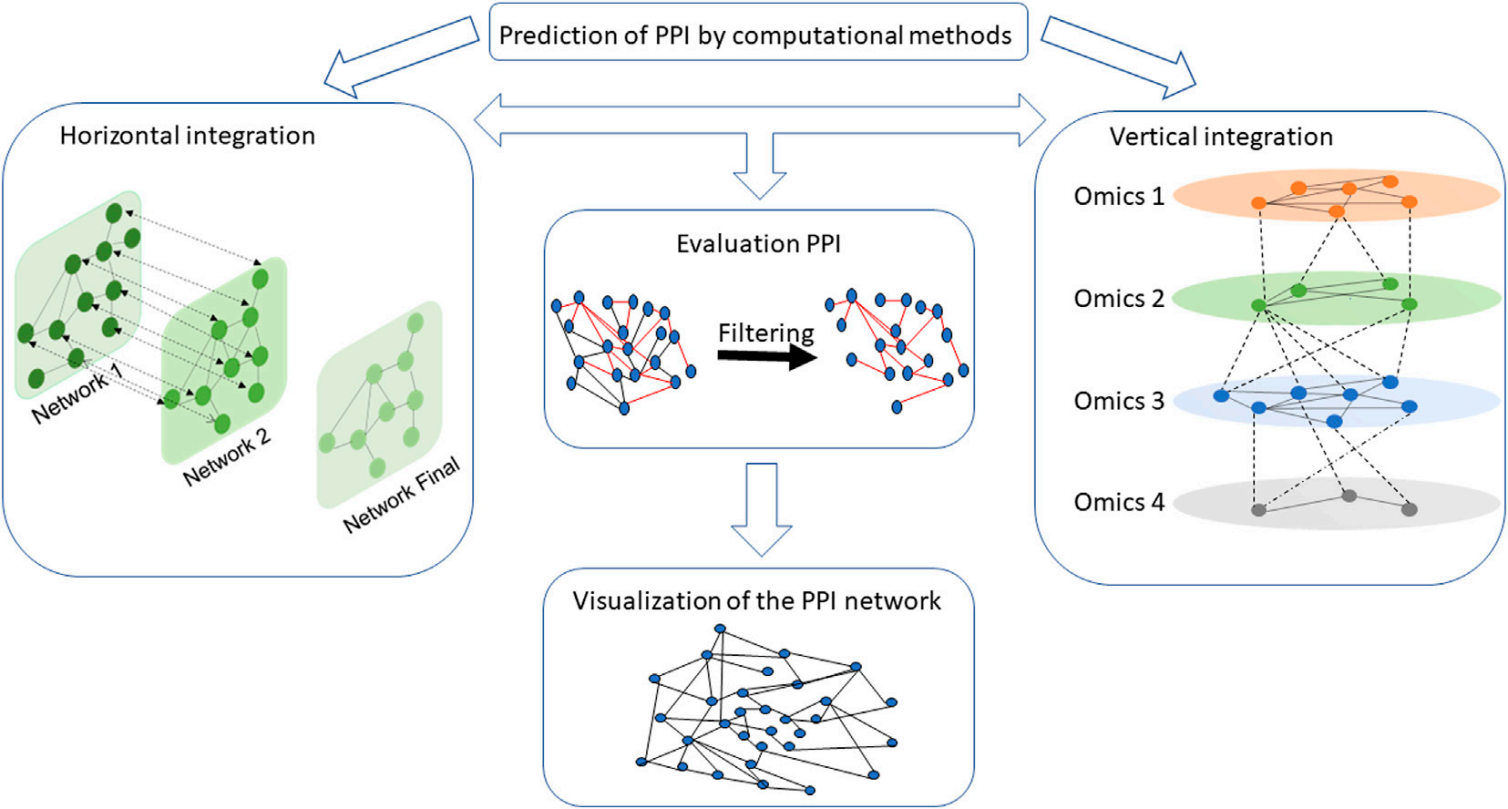
Workflow of key steps to design a PPI network assembly. PPI networks can be integrated horizontally and/or vertically. Horizontal integration creates a PPI network by concatenating interaction information from different PPI databases (here networks 1 and 2 represent two PPI networks from two different databases), while vertical integration gathered information from different omics databases for a given interaction. In the vertical integration box, each omics network represents different interactomes such as protein–protein, drug–protein, and RNA–protein. Once the networks are generated, it is necessary to evaluate its interactions confidence to filter the network. Interactions in red are interactions with a high confidence score. After narrowing the network, specialized tools can be used to visualize the network and information about the connected entities (e.g., identify proteins with a central role in the mechanisms).

A set of interactions between different biological entities that allows the study of biological systems is called an interactome (Cusick et al., 2005; Tieri et al., 2014; Guney et al., 2016; Pinu et al., 2019; Halder et al., 2020; Castillo-Arnemann et al., 2021; Wörheide et al., 2021). Understanding molecular interactions and how they give rise to higher-level functions or diseases is important, especially for repositioning drugs, finding new biomarkers, and potentially developing new therapies or elucidating biological and functional processes (Tieri et al., 2014; Guney et al., 2016; Zhou, Miao and Yuan, 2018; Halder et al., 2020; Castillo-Arnemann et al., 2021; Dimitrakopoulos et al., 2021; L. Wu et al., 2022a). These PPI networks can be integrated horizontally and/or vertically (Lercher and Pál, 2008; Ma and Zhang, 2019). Horizontal integration aimed to create a PPI network from different PPI databases for many interactions (Hibbs et al., 2007; Subramanian et al., 2020), whereas vertical integration will assemble information from different omics (genomics, proteomics, metabolomics, etc.) databases for a given interaction (Wang and Jin, 2017; Ulfenborg, 2019; Das et al., 2020; Welch et al., 2021). All interactions can be modeled into a multi-layered graph structure (Kinsley et al., 2020) where each layer represents a network associated with omic-specific information (Hammoud and Kramer, 2020). PPI networks are a central layer in the multi-omics integration process (Mosca and Milanesi, 2013; Hammoud and Kramer, 2020; Dugourd, Christoph Kuppe and Marco Sciacovelli, 2021) (Figure 1).

Horizontal and vertical integration took advantage of topological properties of the network to facilitate construction of different interactomes, to improve classification and evaluation of a PPI (Peng et al., 2017; Kim, Jeong and Sohn, 2019; Halder et al., 2020; Novkovic et al., 2020). Network topology helps in understanding inter/intracellular interactions and functionality, identifying sub-networks (Banerjee et al., 2020; Pournoor et al., 2020; Mishra, Kumar and Mukhtar, 2021). Thus, the topological properties of a PPI network give insight into dynamics of the network and sub-networks and allow the detection of proteins whose roles can be key in complex central biological mechanisms (Yu et al., 2004; Chen et al., 2019; Wahab Khattak et al., 2021). Filtering the network on topological properties allows the acquisition of highly connected nodes and thus facilitates analysis against the topological data. For example, it is possible to filter network by keeping only proteins of a certain degree (Wu et al., 2009; Navlakha et al., 2014; Azevedo and Moreira-Filho, 2015), or by other topological properties from the graph theory such as, degree distribution (Han et al., 2004; Pablo Porras et al., 2020), shortest path (Du et al., 2014), and transitivity (Hakes et al., 2008; Lynn and Bassett, 2021).

Integration of a PPI network in a multi-omics context is nowadays an essential issue in the understanding of biological mechanisms (Hawe, Theis and Heinig, 2019; Bodein et al., 2021; Dimitrakopoulos et al., 2021). To integrate an interaction into a network, it must first be estimated by a so-called confidence score (Stelzl and Wanker, 2006; Li et al., 2016; Xu et al., 2021), representing probability that the interaction is accurately identified by algorithms and is expressed as a percentage (Kamburov et al., 2012; Peng et al., 2017). This score is usually a ratio of the measured value to the total number of the measured value for each interaction. For example, the Mi-score measures the number of publications observed for an interaction out of the overall number of publications available to the network (Villaveces et al., 2015a). Sub-networks represent a part of the network retaining only interactions with a high confidence score (Flórez et al., 2010; Pietrosemoli and Dobay, 2018; Hao et al., 2019), which can also be extracted to facilitate visualizations. Proteins forming groups called clusters in the sub-networks are recovered. By modifying the threshold of the confidence score, we can better define new clusters and the impact size of the sub-network.

### Horizontal integration of a PPI network

Horizontal integration is a solution to eliminate these false interactions and allows to find missing data, thus adjusting the resulting confidence score (Everson et al., 2019; Gebreyesus et al., 2022). Horizontal integration methods have contributed to development of various types of databases based on organism-specific diseases, biological processes, and detection methods, such as the Integrated Interactions Database (IID) (Kotlyar et al., 2019), IntAct (Hermjakob et al., 2004a), and StringDB (Szklarczyk et al., 2019). PPI is usually redundant in different databases. A PPI found in one database may also be found in others such as BioGRID (Oughtred et al., 2021) or Reactome (Gillespie et al., 2022). This communication between the different databases corresponds to horizontal data integration (Zitnik and Leskovec, 2017; Cowman et al., 2020).

Assembly and merging are the main algorithms for horizontal integrations (De Las Rivas, Alonso-López and Arroyo, 2018; Amanatidou and Dedoussis, 2021). Two PPI networks are assembled by alignment algorithms. Alignment of PPI networks aimed at finding topological and functional similarities between different PPI networks (Kazemi et al., 2016; Ma and Liao, 2020). In a first step, the alignment algorithm looks for overlapping regions in two networks. These regions form clusters that will be assembled to make a local alignment. Then, using local interactions between clusters, a second alignment is performed: global alignment (Malod-Dognin, Ban and Pržulj, 2017; Alcalá et al., 2020; Chow et al., 2021). Other horizontal integration algorithms applied propagation algorithms as the random walk with restart (RWR) process (detailed in vertical integration of a PPI network). Xu et al. (2018) drawled on these propagation methods to reconstruct a multi-level PPI network and identify protein complexes.

Through these different network alignment algorithms, many PPI databases have been updated or created. The most exploited are BioGRID (Oughtred et al., 2021), IntAct (Hermjakob et al., 2004b), String (Szklarczyk et al., 2019), and UniprotKB (The UniProt Consortium, 2019). A large set of databases is referenced in startbioinfo.org (Kshitish et al., 2013) and pathguide.org (Bader, Cary and Sander, 2006). Following the revolution in NGS technology and the increase in PPI datasets, the integration of a single cell with PPI networks is showing promising results. Indeed, the single-cell method coupled PPI network will allow the understanding of gene regulation, cellular heterogeneity (Cha and Lee, 2020), tissue-specific networks, identification of ligand–receptor interactions, functional interactions, and cell–cell communication (Armingol et al., 2021; Johnson et al., 2021; F. Ma et al., 2021a). Cell–cell interactions mediated by ligand–receptor complexes are essential for the coordination of various biological processes, such as development, differentiation, and inflammation. These interactions subsequently ensure that physiological processes are carried out (Vento-Tormo et al., 2018; Efremova et al., 2020). Using single-cell data and PPI networks, it will be possible to understand this crucial interaction and thus to create new therapies targeting these ligand–receptor interactions in future (Ji et al., 2020; Lee et al., 2021). The applications of single cell PPI are numerous and in many fields such as health (Qi et al., 2022) and agronomy (Zhang et al., 2019). These methods will help in the understanding of cellular mechanisms, regulation according to the environment, and in the development of new therapy (Ryu et al., 2019; Mahdessian et al., 2021). Single-cell data can also be used to filter and weight the PPI network following a differential analysis or by filtering according to fluorescence (Dünkler et al., 2015; Wu et al., 2017). Recently, Klimm et al. (2020) have developed SCPPIN, a method of integrating single-cell RNA-seq data with protein–protein interaction networks. By filtering the network by differentially expressed genes and maximum subgraph weight, they detected active modules in cells of different transcriptional states.

However, horizontal integration faces problems such as uniformity of protein interaction identifiers and redundancy of information, data structure, and organization (Dohrmann, Puchin and Singh, 2015; L. Liu et al., 2020a).

### Vertical integration of a PPI network

Vertical integration of networks is generally represented by multi-layer networks (Lv et al., 2021; Watson, Schwartz and Francavilla, 2021). Each layer represents an interactome (protein, gene, and drug). Biological relationships between biological entities and types of interactions form the relationships between different omics layers (Lee and Nam, 2018). Network propagation (or diffusion) algorithms are commonly promoted in omics vertical integration (Di Nanni et al., 2020; Pak et al., 2021). By integrating the information from the different omics and by diffusion algorithms, it is possible to understand the most probable interactions where the diffusion signal has strongly transited (Zhao et al., 2018). Propagation algorithms are a class of algorithms that integrate input data information across connected nodes of a given network. Propagation is usually performed by random walk with restart (RWR) algorithms, inspired by the work of Page et al. (1999) to classify web pages in an objective and mechanical way. RWR is the state-of-the-art approach to infer the relationship: as the name suggests, a random walker, starting from a set of nodes of interest (starting nodes), jumps to neighboring nodes, or nodes in another layer according to a certain probability assigned to the edges of the nodes (Lee and Yoon, 2018). In addition, the walker has a certain probability, known as the damping factor, such that for each step taken in any direction, there is a probability associated with returning to one of the original sets of nodes (Valdeolivas et al., 2019; Nguyen et al., 2021; Qu et al., 2021; Wen et al., 2021). The probability is calculated from a transition matrix from one node to the other, allowing to obtain a weight for each interaction. This node-dependent weight will reflect an interaction between two omics layers (Bhatia, 2019; Dupré, 2022). Lei et al. (2019a) adjusted this method to detect essential proteins. In this method, PPIs are weighted according to network topology, gene expression, and GO annotation data. Then, an initial score is assigned to each protein in a PPI network by exploiting information on subcellular localization and protein complexes. Then the RWR algorithm is applied to the weighted PPI networks to iteratively score the proteins, allowing the filtration of interactions with high weight.

The main other algorithms based on topological properties use integration strategies from two classes: empirical methods and machine learning method (Jin et al., 2014; Haas et al., 2017; Eicher et al., 2020). Empirical methods simply assembled different layers of the network, whereas machine learning methods tried to find missing information about how information flows between the omics layers (Picard et al., 2021; Santiago-Rodriguez and Hollister, 2021). MoGCN (Li X. et al., 2022) is a tool for multi-omics integration based on a convolutional graph network. This tool allows the classification and analysis of cancer subtypes. MoGCN can extract the most significant topological features and properties of each omic layer for downstream biological knowledge discovery.

Integration of PPI networks into multi-layer networks has a central role (Liang et al., 2019; Huang and Zitnik, 2021). Indeed, projection of PPI and layer connectivity allows improvement of the mechanistic and functional knowledge of a cell, identifying key proteins and repositioning drugs (F. Li et al., 2020c). Silverbush and Sharan (2019) created an approach to direct the human PPI network using the drug response and cancer genomic data. A directed graph is a graph in which the edges have a direction. The direction of the relationships or edges is found by diffusion methods. The oriented network allows the detection of key genes in cancers.

In vertical or horizontal integration, the PPI layer must be reliable. The topological properties of the network can allow the establishment of a confidence score for a given interaction. It is essential to understand these properties to build the most robust network possible (Zhang, Xu and Xiao, 2013; Sardiu et al., 2019).

### Validation of PPI

An important question persists in network analysis: can we trust on the network of interactions to be a true biological interaction? PPIs from these methods have supplied insights into functions of individual proteins, regulatory pathways, molecular mechanisms, and entire biological systems. Noise inherent in the interactome information hinders evaluation of PPI data (Correia et al., 2019). Several PPIs are, in fact, false positives in these methods and even in methods using strict criteria to define a positive (Yu et al., 2004; Scott and Barton, 2007). It should be noted that the coverage of the interactome is also incomplete and uneven, so we cannot always filter out the less reliable evidence (Han et al., 2005; Stelzl and Wanker, 2006). Many different methods exist for finding reliability and giving a measure of confidence. These techniques can be classified into three main categories.

#### Contextual biological information

This strategy for assessing the veracity of an interaction looked for different information, for example, overlapping patterns of co-expression, conservation of structure, and sequences (Aytuna, Gursoy and Keskin, 2005; Tirosh and Barkai, 2005). As an example, Schaefer et al. (2013) seek biological information based on influenza virus knowledge to validate PPIs.

#### Scores based on the literature

Acts as an orthogonal validation and analyzed how often a PPI is cited in publications. The main problem with implementing this method is the application of thresholds, so that only interactions with a sufficiently high score are retained (Bozhilova et al., 2019). Well-studied proteins will have a greater number of interactions and associated publications than proteins that are new or have little information. Hence, thresholds need to be standardized. In order to normalize thresholds among different databases, the MI-score method was created (Villaveces et al., 2015a). This method allows to merge data from different databases that are in the PSI–MI(Proteomics Standards Initiative–Molecular Interaction) format (Hermjakob et al., 2004a; Bader, et al., 2006; Kerrien et al., 2007), and link an interaction to a notation system. This method generates three different scores: publication score (number of different publications on an interaction), method score (considers the different methods of detecting an interaction), and the type of score which refers to the type of interaction. The type of interaction follows the nomenclature of the PSI-MI controlled vocabulary, for example, genetic interaction, physical association, and co-location.

#### Aggregated methods

Use different score calculation strategies and combine these strategies into a single score. Several scoring methods exist, including the toolkit developed by Braun et al. (2009) that includes four statistical tests to verify a PPI from a high-throughput experiment. The results of the four tests are then combined to calculate the probability that a new pair of interactions is a true biophysical interaction. Intscore is a reference aggregation tool, which calculates confidence scores for user-specified sets of interactions. Its scoring system is based on network topology and annotations. The aggregated score can be computed by machine learning approaches (Kamburov et al., 2012). Recently, Paul and Anand (2022) developed several similarity measures using GO to create a confidence score for PPIs.

Apart from these three distinct categories, to measure the confidence of PPIs, robust measures resulting from data provenance and network topology are needed, such as the average redundancy difference between various sources, natural connectivity of the PPI network as well as the number of edges in a protein-centered sub-arrays (ego networks) (Bozhilova et al., 2019; Wang et al., 2019). The main problem with all these methods is that a score is mainly specific to one database, so threshold values are highly database dependent (Kamburov et al., 2012; Dahiya et al., 2019; Xu et al., 2019). To address this issue, consensus networks appeared such as HugGan (Huang et al., 2022) which is a tool that gathers 31 data sources using deep learning approaches to keep only interactions with a high confidence score resulting in a network with high coverage and quality.

### Visualization of protein–protein networks

Networks are a powerful way to visualize complex systems (Charitou, Bryan and Lynn, 2016; Mlecnik, Galon and Bindea, 2018). Visualization of PPI networks is crucial for the understanding of pathways, sub-graphs, sub-network, and central proteins (Sharan and Ideker, 2006; Fionda et al., 2009; Snider et al., 2015; De Las Rivas, Alonso-López and Arroyo, 2018; Vella et al., 2018; Marai et al., 2019). The simplistic and rapid visualization of networks makes it a tool of choice (Gillis, Ballouz and Pavlidis, 2014; Chung et al., 2015; Xu et al., 2021). This has led to the development of methods and tools that allow visualization. The integration of PPI networks and their visualizations in a multi-omics context has helped in the modeling of complex systems such as Parkinson’s disease (Tomkins and Manzoni, 2021), identifying central proteins in diseases (Narayanan et al., 2011; Deng, Xu and Wang, 2019), understanding protein clusters linked to cellular function (Zhao, Wang and Wu, 2017; Amanatidou and Dedoussis, 2021), understanding mechanisms of action (Jia et al., 2021; Yuan et al., 2021), and drug repositioning (Lee and Yoon, 2018; Soleimani Zakeri, Pashazadeh and MotieGhader, 2021).

Larger and complex networks are more difficult to visualize. This is the case of the most popular source offering a representation of PPI networks such as StringDB (Szklarczyk et al., 2019). This online database is intended for the inspection of small networks or sub-networks (less than 500 interactions). Therefore, because of their size and topology, the PPI network requires specialized tools (Bosque et al., 2014; Freilich et al., 2018; Aihaiti et al., 2021).

The methods for visualizing a network can be divided into three categories (Table 2).

**TABLE 2:** Summary table of tool for visualizing of protein–protein interaction network. Visualization methods to analyze network are grouped into three distinct categories: visualization through downloadable tools, visualization by libraries integrated with languages, and visualization through graph-oriented databases. The user has to choose his tools according to his study context. For analysis of high dimensional data containing a large amount of information, it is advisable to manipulate tools based on graph databases. Conversely, if the user wants to have a quick representation, we recommend the user to turn more to visualization libraries or downloadable software.

	Tool	Advantage	Disadvantage
Visualization through downloadable tools	Cytoscape (Otasek et al., 2019), Gephi (Bastian, Heymann and Jacomy, 2009), Tulip (Auber et al., 2017), Graphviz (Ellson et al., 2001), Pajek (Mrvar and Batagelj, 2016)	Many add-on features, flexibility for network analysis, easy to handle, open source and free	Difficult to set up automation interface, working with big networks requires big memory and computing power
Visualization by libraries integrated with languages	Igraph (Csárdi and Nepusz, 2006), NetworkX (Hagberg et al., 2008), graph-tool (Peixoto Tiago, 2014), NetView (Neuditschko, Khatkar and Raadsma, 2012)	Open source and free, well documented, accessible, import and export graphs easily, easy to implement	Graphic possibilities are limited, restricted number of nodes
Visualization through graph-oriented databases	Neo4j (Gong et al., 2018), ArangoDB (*ArangoDB NoSQL Multi-Model Database: Graph, Document, Key/Value*, 2022), JanusGraph (Sharp. 2017), OrientDB (Tesoriero, 2013), Elasticsearch (Shay Banon, 2014), Siren (Giovanni Tummarello and Renaud, 2015)	Speed of calculation, adapted big networks, integrated search engine, Flexible and agile structures	Request for calculation servers. Not very scalable as it is designed for a single server architecture

The methods can be combined to take advantage of each of the benefits of these categories. This is the case with cyNeo4j (Summer et al., 2015) which combines Cytoscape (Otasek et al., 2019) and Neo4j (Gong et al., 2018) for fast visualization of large networks based on a graph-oriented database. Cytoscape is the most widely used tool for the visualization of large networks (Shannon et al., 2003). Other visualization systems do not fit into these categories and are based on web-based visualization interfaces and on a relational database (Salazar-Ciudad and Jernvall, 2013; Salazar et al., 2014; Hayashi et al., 2018). This is the case of the PINA 3.0 (Du et al., 2021) tool, which is a consensus database containing five interactomes and offering a web visualization service allowing the identification of interacting protein pairs in different cancer types. The weaknesses of these methods are the size of the networks, the execution time of a query, and their limited applicability (Jeanquartier, Jean-Quartier and Holzinger, 2015; Zhou and Xia, 2018; Perlasca et al., 2020).

Visualization tools are evaluated by four criteria: compatibility (available on which OS (operating systems): Windows, Mac Os, and Linux, analytic functions (presence of functions measuring the topological properties of the network, weak interactions of external data, etc.), visualizations (graph layout, dynamics, and parallel implementation), and the extensibility of the tool (addition of plugins, type of input, and output file) forming distinct classes (Sanz-Pamplona et al., 2012; Agapito, Guzzi and Cannataro, 2013; Dallago et al., 2020). In the context of biological network analysis and in particular protein networks, one of the essential criteria is dynamic visualization tools (Xia, Benner and Hancock, 2014; Zhou and Xia, 2018). PPI networks have a dynamic organization of biological sub-networks (Yang, Wagner and Beli, 2015). In other words, the molecular interactions in a cell vary in time, as do the signals from the environment surrounding an interaction (Przytycka, Singh and Slonim, 2010; M. Li et al., 2018b).

In order to overcome the limitation of network size and consider the dynamics of the networks, several tools have been developed over the last decades (Sanz-Pamplona et al., 2012; Winkler et al., 2021). The success of Cytoscape is due to the large number of plugins/features that can be added directly from the tool (Saito et al., 2012; Lotia et al., 2013). The calculation of overrepresented GO terms in a network can be performed by Bingo (Maere, Heymans and Kuiper, 2005), a widely downloaded Cystoscape plugin. Through Cytoscape, we also find plugins allowing the understanding of the dynamic organization of biological networks such as TVNViewer (Curtis et al., 2011), KDDN (Tian et al., 2015), and Dynetviewer. Another downloadable software offering a visual representation of PPI networks is the Gephi (Bastian, Heymann and Jacomy, 2009). Downloadable network visualization tools have difficulties with the implementation of data (Villaveces et al., 2015b; Li et al., 2016). Visualization libraries such as igraph (Csárdi and Nepusz, 2006) and NetworkX (Hagberg et al., 2008) will make it easier to import and export networks but are limited in terms of adding new functionality and graphic possibilities (Pandey, 2018; L. Wu et al., 2022a).

Network visualization tools are specific to the detection method (Ashtiani et al., 2018). HPIminer (Subramani et al., 2015) extracts information from human PPIs and PPI pairs in biomedical literature and provides a visualization of interactions, networks, and associated pathways using two databases, namely, HPRD (Goel et al., 2012) and KEGG (Kanehisa et al., 2016). Another area of improvement for online or general-purpose visualization tools and libraries is the addition of a visualization engine or search engine (Chisanga et al., 2017). Tools integrating visualization engines such as NAViGaTOR (Brown et al., 2009) and MIST (Hu et al., 2018) have been developed. These tools allow the acceleration of the visualization of large PPI networks (Yu and Zhang, 2008; Gerasch et al., 2014; Zaki and Tennakoon, 2017). It is also possible to improve the speed of visualizations by connecting directly to graph databases such as Neo4j (Gong et al., 2018, p. 4) and ArangoDB (Touré et al., 2016; Timón-Reina, Rincón and Martínez-Tomás, 2021; *ArangoDB NoSQL Multi-Model Database: Graph, Document, Key/Value*, 2022). Since graph databases store data directly in a graph form, they are becoming a preferred resource for storing complex relationships of heterogeneous biological data (Yoon, Kim and Kim, 2017; Jupe et al., 2018; Castillo-Arnemann et al., 2021). Flexibility of multi-omics integration offered by graph databases facilitates data mining to support different hypotheses (Lysenko et al., 2016; Brandizi et al., 2018; Wandy and Daly, 2021).

All these tools for the visualization of PPI networks are based on different visualization algorithms (Koutrouli et al., 2020; Sandoval and Orlando, 2021). Visualization algorithms can be based on simplistic approaches such as adjacent matrices (Fekete, 2009), circular layouts (Suderman and Hallett, 2007), or complex approaches such as force-directed algorithms (Liu et al., 2021). The main differences between simple and complex algorithms for visualization depend on the size of the network, the topology of the network, and the dimensionality of the information (Heberle et al., 2017; Becker et al., 2020; Raja et al., 2020). The selection of the appropriate visualization algorithm will depend on the nature of the network. In the context of single networks, in particular PPI networks, visualization algorithms focus on the identification of protein sub-clusters or hub proteins (Li et al., 2020b; H. Ma et al., 2021b). Cytoscape’s Cytohubba (Chin et al., 2014) plugin is commonly dedicated for sub-network identification and central protein identification. The most powerful method of Cytohubba for better sub-network visualization is the maximum clique centrality (MCC) method. This algorithm allows the visualization of groups of proteins called clusters, based on the assumption that essential proteins tend to be grouped together (Lu et al., 2010; Lei et al., 2019b; Kim, Jeong and Sohn, 2019). Recently, Zu et al. (2017) used this plugin’s method to visualize six target genes for quercetin (an organic compound of the flavonoid family), suggesting a therapeutic potential in type 2 diabetes mellitus (T2DM) and Alzheimer’s disease.

However, in a multi-omics integrations context one seeks above all to connect information from different omics fields (transcriptomics, proteomics, metabolomics, lipidomics, and metabolomics (Haas et al., 2017; Fan, Zhou and Ressom, 2020; Cansu Demirel, Kaan Arici and Tuncbag, 2022). In this context, multi-layer algorithms for visualization are preferable to force-directed algorithms (Bodein et al., 2021; Dursun, Kwitek and Bozdag, 2021; Marín-Llaó et al., 2021). There are several algorithms for implementing multi-layer networks, in the context of multi-omics integration, the most highlighted implementation is the one named by Hammoud and Kramer, (2020): “Interactive/Interconnected/Interdependent Networks and Networks of Networks Implementation.” This implementation has as input a set of monoplex networks (single layer networks, e.g., PPI network). Each network interacts with the other networks. The different monoplex networks will form distinct layers which will be connected by the inter-side nodes (Rappoport and Shamir, 2018; Yan et al., 2018; Zoppi et al., 2021; Cuenca et al., 2022). Recently Arena3dweb (Karatzas et al., 2021), a web application incorporating these algorithms and offering a visualization of multi-layer graphs in a 3D space, has enabled GPCR signaling pathways implicated in melanoma.

## Summary and outlook

In this review, different computational strategies for predicting PPI, from integration to visualization to methods for validating interactions have been studied. Many computational prediction approaches rely on experimental methods to predict a PPI interaction (Rao et al., 2014; Peng et al., 2017; Ding and Kihara, 2018; Tanwar and George Priya Doss, 2018). Although this increases the coverage of the network, it can disrupt the horizontal integration process (Browne et al., 2010; Ngounou Wetie et al., 2013). Sets of PPI interactions from different datasets are constructed and transformed independently, which can lead to information gaps, redundant information, and poor identifier compatibility when aligning two PPI networks. Ideally, at any point in the overall integration process (including vertical and horizontal), each omics data set should be evaluated in the context of the other datasets, so that complementary information can be fully exploited, and added information can be identified (Bozhilova et al., 2019; Bajpai et al., 2020). Implementation of validation scores based on topological properties allows to limit the redundancy of edges and will allow to filter the PPI network (Pietrosemoli and Dobay, 2018; Sardiu et al., 2019).

Information redundancy is the repetition of information without adding additional information in different databases. The increase in omics data and PPI integration methods has contributed to the growth of many PPI databases. However, this increase in the number of databases increases the redundancy of information, making it difficult for the user to choose a PPI database (Rabbani et al., 2018; Hawe, Theis and Heinig, 2019; Zahiri et al., 2020). In addition, information redundancy slows down the calculation time for the construction and visualization of networks (Chen et al., 2019, 2019). To limit and remove redundancy, different information scores have been set up (Silverbush and Sharan, 2019; Mahdipour and Ghasemzadeh, 2021). The Mi-score (Villaveces et al., 2015b) consisting of three scores, is increasingly used to validate a PPI.

The study of PPI networks is a growing field of systems biology. Due to their significant role, PPI networks are used to understand cellular functions or biological mechanisms (Stelzl and Wanker, 2006; Jordán, Nguyen and Liu, 2012; Safari-Alighiarloo et al., 2014). The integration of these networks, both vertically and horizontally, can highlight clusters of proteins with central roles, aiding the understanding of drug action mechanisms (Martin, Roe and Faulon, 2005; Dimitrakopoulos et al., 2021; Marín-Llaó et al., 2021; Tomkins and Manzoni, 2021). PPI networks offer prospects in many fields, such as medicine, health and also in agri-food (Hao et al., 2019; Hasan et al., 2020; Thanasomboon et al., 2020; Charmpi et al., 2021). Vertical and horizontal integration algorithms are mainly based on propagation and alignment algorithms but are often combined with machine learning methods to predict the probability of reliability of an interaction (Li and Ilie, 2017; Lee and Nam, 2018; Zhang et al., 2018; Das and Chakrabarti, 2021). These propagation algorithms will allow to focus on sub-networks, keeping only the interactions where the propagation signal is high (Gehlenborg et al., 2010; Laniau, 2017).

By focusing on sub-networks as opposed to complete networks, visualization is facilitated allowing the identification of sub-groups of interactions (Tian, Ju and Yang, 2019; T.-H. Liu et al., 2020b). The visualization of networks is a problematic issue for networks and especially for PPI networks (Du et al., 2021). Visualization tools depend mainly on the size of our networks (Summer et al., 2015; Zou et al., 2017). Currently, multilayer network visualization is limited to small networks and requires a consequent pre-formatting of the data (Smith-Aguilar et al., 2019; Hammoud and Kramer, 2020; Sebestyén, Domokos and Abonyi, 2020).The study of multilayer networks based on the PPI network is constantly evolving and will become more powerful with advancement of more powerful mathematical models offering better predictions (Kapadia et al., 2019; Karatzas et al., 2021; Cuenca et al., 2022). Different perspectives on the integration of PPI networks can be imagined. The visualization of multilayer multi-omics networks and creation of consensus networks for each omics dimension to understanding new mechanisms of multi-omics integration. A consensus network is the result of the horizontal integration of different databases (Berto et al., 2016; Mosca et al., 2021). Through this network, it will be possible to homogenize the different thresholds of the different databases and to eliminate the recurrence of information (Leblanc et al., 2013; Affeldt et al., 2016; Zohra Smaili et al., 2021). Recently, Woo and Yoon (2021) created a Monaco aligner that can find multiple alignments with high accuracy to identify functional modules. In the era of big data and NGS (next generating sequencing) technologies, it is difficult to know which information is needed to build a PPI network. Machine learning and deep learning methods offer novel perspectives in the prediction and standardization of information in PPI networks (Gligorijević and Pržulj, 2015; Borhani et al., 2022; Cervantes-Gracia, Chahwan and Husi, 2022). Standardizing and evaluating the relevance of interactions will facilitate integration of PPI networks (Fiorentino et al., 2021; Nadeau, Byvsheva and Lavallée-Adam, 2021).

On the visualization side, several perspectives can be imagined, a tool to visualize each layer independently and globally in a multilayer network (Kanai, Maeda and Okada, 2018; McGee et al., 2019). As the size and complexity of PPI networks increases, more efficient visualization algorithms are needed (Chong, Wishart and Xia, 2019; Koutrouli et al., 2020). Augmented reality technologies and virtual reality (VR) remove the constraints of 2D/3D space constraints (Pirch et al., 2021; Hütter et al., 2022). Moreover, the notable advances in the prediction of the structure of proteins from their sequence in amino acids with alphafold (Jumper et al., 2021), which could lead to a revolution in the PPI prediction algorithm. In view of the generous size of PPI networks, visualization tools focus on specific networks, including Mechnetor (González-Sánchez et al., 2021), a tool for visualization of biological mechanisms. At the moment, there are no tools available to visualize the interactome protein specific to a tissue, but there are different databases on this subject (Islam et al., 2013; Basha et al., 2018).
